# Menstrual, reproductive, and contraceptive factors associated with cervical cancer in women aged ≤35 years: an individually matched case–control study

**DOI:** 10.3389/fonc.2026.1931698

**Published:** 2026-09-22

**Authors:** Lian Liu, Yuying Zhang, Limin He, Lihua Zhou

**Affiliations:** 1 Department of Very Important Person (VIP) clinic Nursing, West China Second University Hospital, Sichuan University/West China School of Nursing, Sichuan University, Chengdu, Sichuan, China; 2 Key Laboratory of Birth Defects and Related Diseases of Women and Children, Sichuan University, Ministry of Education, Chengdu, Sichuan, China

**Keywords:** associated factors, case-control study, cervical cancer, menstruation, reproductive history

## Abstract

**Objective:**

This study aims to investigate the associations of menstrual, reproductive, and contraceptive characteristics with early-onset cervical cancer among women aged ≤35 years.

**Methods:**

A hospital-based, individually matched case–control study was conducted at West China Second University Hospital from January 2022 to December 2024. A total of 300 women aged ≤35 years and with histologically confirmed cervical cancer were individually matched 1:1 to 300 screening controls within 0.5 years of age and 7 days of recruitment. Menstrual, reproductive, and contraceptive histories accrued up to 90 days before the index data were collected through standardized interviews and medical record reviews. Separate exposure-specific conditional logistic regression models stratified by matched pair were adjusted for prespecified sociodemographic, behavioral, screening-related, infection-related, and current high-risk human papillomavirus (hrHPV) covariates. The model samples ranged from 189 to 277 matched pairs. Exploratory subgroup analyses assessed effect modification.

**Results:**

In the fully adjusted model, menstrual period duration was associated with early-onset cervical cancer, with evidence of nonlinearity (*P* for nonlinearity <0.001). Each 1-year increase in age at first pregnancy was associated with lower odds (adjusted odds ratio [aOR], 0.717; 95% confidence interval [CI], 0.561–0.917). Higher odds were associated with greater gravidity (aOR per pregnancy, 2.959; 95% CI, 1.734–5.050), greater parity (aOR per birth, 3.667; 95% CI, 1.886–7.133), and more induced abortions (aOR per event, 2.672; 95% CI, 1.236–5.773). Longer total breastfeeding duration was inversely associated (aOR per 12 months, 0.189; 95% CI, 0.064–0.560), whereas longer cumulative combined oral contraceptive use was positively associated (aOR per 12 months, 1.335; 95% CI, 1.062–1.678). Age at menarche, menstrual cycle irregularity, ever breastfeeding, categorical combined oral contraceptive status, and other contraceptive methods were not statistically significant after full adjustment. No interaction remained statistically significant after false discovery rate correction.

**Conclusions:**

Selected menstrual, reproductive, and contraceptive characteristics were associated with early-onset cervical cancer after adjustment for measured confounders and current hrHPV status.

## 1 Introduction

Cervical cancer remains a significant global health burden, ranking as the fourth most common cancer among women worldwide (1). In 2022, the global age-standardized incidence rate (ASIR) and age-standardized mortality rate (ASMR) were 14.12 and 7.08 per 100,000 women, respectively (1). The overall incidence of cervical cancer has declined in many high-income countries, coinciding with the implementation of cervical screening and human papillomavirus (HPV) vaccination programs (2, 3). However, cervical cancer occurring in younger women remains clinically important because its diagnosis and treatment may coincide with key reproductive years (1, 4–7).

Early-onset cervical cancer may have clinicopathological characteristics that differ from those observed in older women. Young women have shown differences in histological subtype, tumor differentiation, stage, and lymph node involvement across observational studies, although the findings have not been uniform (5, 6, 8, 9). Moreover, the diagnosis of early-onset cervical cancer has implications for fertility preservation, reproductive health, and long-term quality of life (5–7). Therefore, focusing on women aged ≤35 years may help clarify factors associated with cervical cancer in this age group. While persistent high-risk HPV (hrHPV) infection is the central etiological factor in cervical carcinogenesis, epidemiological evidence suggests that hormonal and reproductive characteristics may also be associated with cervical cancer, particularly among younger women (10–12). Menstrual characteristics may reflect variation in reproductive and hormonal exposures. A previous database study reported an association between menstrual irregularities and cervical cancer diagnosis (13). Menstrual hygiene practices have also been examined in relation to cervical precancer and cervical cancer, although the available evidence remains limited (13, 14). Similarly, reproductive factors such as parity, breastfeeding history, and contraceptive use have been associated with cervical cancer in observational studies, although the reported associations have varied (10, 11, 15–17).

Previous studies investigating menstrual and reproductive factors in cervical cancer have predominantly focused on mixed-age populations or older women, with limited data specifically addressing women aged ≤35 years. Therefore, this study conducted an individually matched hospital-based case–control study to evaluate the association between menstrual, reproductive, and contraceptive characteristics and early-onset cervical cancer in Chinese women aged ≤35 years. This study aimed to estimate adjusted associations while accounting for prespecified sociodemographic, behavioral, screening-related, infection-related, and current hrHPV covariates.

## 2 Materials and methods

### 2.1 Study design

This hospital-based, individually matched case–control study evaluated associations between menstrual and reproductive factors and early-onset cervical cancer among women aged ≤35 years. The study was conducted at West China Second University Hospital, Sichuan University, from January 2022 to December 2024. The analytic sample comprised 300 individually matched case–control pairs. Each control was matched to a case within 0.5 years of age and 7 days of recruitment, and pair identifiers were retained for analysis. The index date was the date of histologic diagnosis for cases and the corresponding cervical screening date for controls. Exposures were defined using information accrued up to 90 days before the index date.

All study procedures were conducted in accordance with the Declaration of Helsinki. The study protocol, including participant recruitment, face-to-face interviews, standardized questionnaire administration, and written informed consent procedures, was approved by the Institutional Review Board of West China Second University Hospital, Sichuan University on December 6, 2021 (approval no. 2021562061214), before participant recruitment and interviewing began. All participants provided written informed consent before the standardized interview.

### 2.2 Patient selection

Eligible participants were women aged ≤35 years who received cervical screening, diagnostic evaluation, or treatment at the same hospital during the study period. Cases were women with histologically confirmed invasive cervical cancer, including screen-detected, symptomatically diagnosed, and incidentally detected disease. Eligibility required a pathology report documenting invasive cervical carcinoma; histologic subtype and International Federation of Gynecology and Obstetrics (FIGO) stage were abstracted from diagnostic records (18).

Controls were individually matched women from the same screening population with cytology classified as negative for intraepithelial lesion or malignancy (NILM) at the index examination; high-risk HPV positivity alone did not preclude control eligibility. Women with atypical squamous cells of undetermined significance or worse cytology, cervical intraepithelial neoplasia grade 2 or higher, or cervical cancer at the index evaluation were excluded. HPV-positive/NILM controls underwent repeat screening 6–18 months after the index date, with colposcopy and biopsy performed when clinically indicated; control eligibility required no diagnosis of cervical intraepithelial neoplasia grade 2 or higher or cervical cancer during follow-up. Both cases and controls were excluded if they were pregnant at the time of enrollment, had severe psychiatric disorders that would impair their ability to provide reliable information, or had incomplete medical records or missing data regarding menstrual and reproductive history. Core menstrual and reproductive variables were required for both members of each pair and were cross-checked between medical records and standardized interviews conducted by trained nurses. The workflow of patient selection is shown in **Figure 1**.

**Figure 1:**
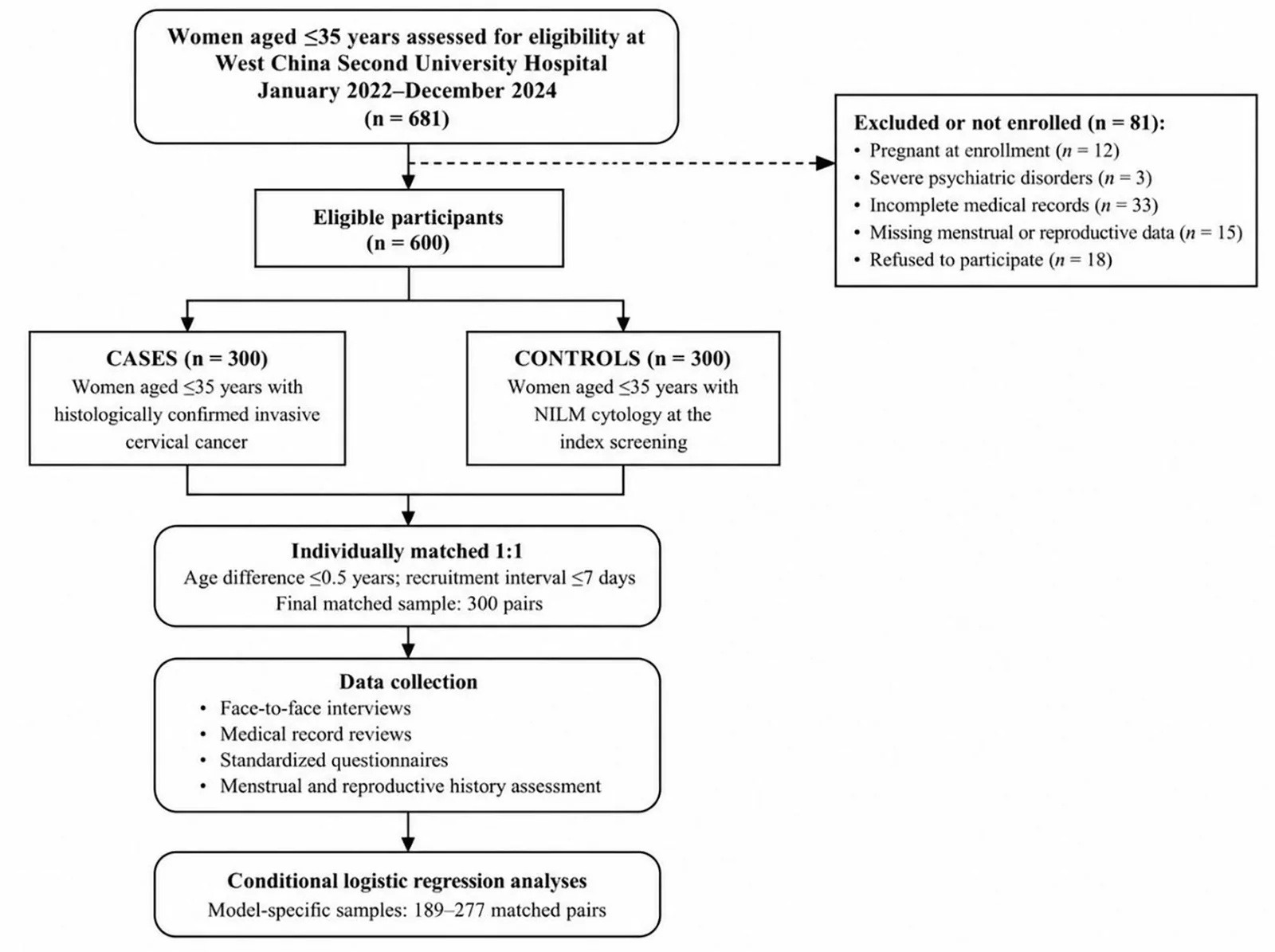
Flow diagram of participant assessment, exclusion, individual matching, and analysis. The diagram summarizes the assessment of 681 women, the exclusion or non-enrollment of 81 women, and the final inclusion of 300 women with histologically confirmed early-onset cervical cancer and 300 screening controls individually matched by age and recruitment time.

### 2.3 Data collection and variable definitions

Data were collected using face-to-face interviews, medical record reviews, and standardized questionnaires.

Standardized interviews were administered by trained nurses at recruitment, except for a subset of cases interviewed within 5 days after histologic diagnosis; diagnosis-awareness status at interview was recorded. Only information occurring on or before the exposure cutoff date was included in exposure definitions. Interview data were cross-checked against source records and routine clinical documentation.

Demographic and prespecified covariate data included age at the index date, body mass index (BMI), education, occupation classified according to the International Standard Classification of Occupations, household income, residence type, cervical screening frequency during the previous 5 years, HPV vaccination, sexual history, smoking, and chlamydial infection history. Menstrual exposures included age at menarche in completed years, average cycle length and menstrual period duration in days, menstrual flow assessed with a standardized pictorial blood loss assessment chart and categorized as light, normal, or heavy, and cycle regularity during the 12 months before the exposure cutoff (19). Cycle variation of less than 7 days was classified as regular.

Reproductive variables included ever pregnancy, age at first pregnancy, gravidity, parity, spontaneous abortions, medication-induced abortions, and surgical abortions. Medication-induced and surgical abortions were summed for the induced-abortion measure, whereas spontaneous and induced abortions were analyzed separately. Breastfeeding variables comprised ever breastfeeding and cumulative duration in months among women with at least one birth. Contraceptive exposures distinguished combined oral contraceptives from progestin-only pills; combined oral contraceptive status was classified as never, current, discontinued within 12 months, or discontinued more than 12 months previously. Cumulative duration was recorded in months. Copper intrauterine devices, levonorgestrel-releasing intrauterine systems, injectable contraceptives, contraceptive implants, and barrier methods were recorded separately. Sexual behavior variables included age at first sexual intercourse, lifetime number of sexual partners, number of new partners during the previous 12 months, and high-risk behavior in partners. Smoking was classified as never, former, or current, and passive smoking was recorded separately. Laboratory and clinical records also provided chlamydial infection history, human immunodeficiency virus status, pathology review, and tissue HPV confirmation when available. Current hrHPV-negative cases were further characterized by histologic type, pathology review status, tissue HPV confirmation, and mode of detection. Current hrHPV-positive/NILM controls were characterized by repeat screening, colposcopy, biopsy, and final follow-up status. HPV16 and HPV18 results were summarized by histologic type among cases.

### 2.4 HPV assessment

High-risk HPV status was based on the result closest to the index date, obtained from clinician-collected cervical exfoliated cells from 21 days before through 7 days after the index date. Testing used the Cobas 4800 HPV DNA, Hybribio 14-type high-risk HPV DNA, or Sansure 15-type high-risk HPV DNA assay. Assay-specific results were harmonized as current hrHPV-positive or hrHPV-negative. Genotype-specific analyses were restricted to HPV16 and HPV18; HPV52 and HPV58 were not included. Persistent high-risk HPV was defined as positive results on two tests at least 6 months apart when a prior result was available (20). Model 2 estimated associations without HPV adjustment, and model 3 included current hrHPV status as a prespecified infection-related covariate based on its established role in cervical carcinogenesis.

### 2.5 Subgroup and sensitivity analyses

Exploratory subgroup analyses were specified for five characteristics. The subgroups were defined as follows (1): educational level (primary education or below vs. secondary education and above), (2) occupation type (manual labor vs. professional/managerial and clerical/service combined), (3) age at first sexual intercourse (≤18 years vs. >18 years), (4) smoking status (never smokers vs. ever smokers including former and current smokers), and (5) HPV infection status (positive vs. negative). The examined exposures were age at menarche, irregular menstrual cycles, spontaneous abortions, induced abortions, and cumulative combined oral contraceptive use. For each exposure and subgroup factor, a conditional logistic model included the exposure, subgroup indicator, and exposure-by-subgroup interaction, together with the model 3 covariates; the subgroup main-effect term represented that factor when it was also among the model 3 covariates. Subgroup-specific estimates were obtained as linear combinations, and interaction was evaluated using a likelihood-ratio test. Benjamini–Hochberg false-discovery-rate correction was applied across the 25 interaction tests. Sensitivity analyses retained pairs with unknown household income using the median ordinal income value and an unknown-income indicator; added BMI, new partners in the previous 12 months, high-risk partner status, passive smoking, other sexually transmitted infection history, and immunosuppression to the adjustment set; restricted analyses to pairs with hrHPV-positive cases, pairs in which both members were current hrHPV-positive, or common histologic types; excluded the most influential matched pair; excluded the nine hrHPV-positive/NILM controls lost to follow-up and their matched cases; and excluded cases interviewed after diagnosis and their matched controls. The analysis restricted to pairs with hrHPV-positive cases used the model 2 covariates, whereas the analysis restricted to pairs in which both members were hrHPV-positive adjusted for education, smoking status, age at first sexual intercourse, lifetime number of sexual partners, and cervical screening frequency.

### 2.6 Statistical analysis

Statistical analyses were performed in Python 3.12.13. pandas 3.0.1 was used for data management, while conditional logistic regression, restricted cubic splines, interaction testing, Benjamini–Hochberg false-discovery-rate correction, and model diagnostics were implemented using custom NumPy 2.3.5 routines. The conditional likelihood for complete 1:1 matched pairs was maximized by Newton–Raphson iteration. Tests were two-sided, with *P <*0.05 considered statistically significant. Continuous variables were presented as means ± standard deviations (SD) or medians with interquartile ranges (IQR). Categorical variables were described as frequencies and percentages (%). Matched-pair comparisons used paired *t*-tests for approximately normally distributed continuous variables, Wilcoxon signed-rank tests for skewed or count variables, McNemar or exact McNemar tests for binary variables, and Bowker symmetry tests for multinomial variables. The adjustment set was prespecified before model fitting on clinical and epidemiological grounds (21–24). Each exposure was evaluated in a separate conditional logistic regression model conditioned on the individually matched case–control pairs.

Model 1 included the exposure alone and estimated the unadjusted matched association. Model 2 additionally included education, occupation, household income, residence type, smoking status, age at first sexual intercourse, lifetime number of sexual partners, cervical screening frequency during the previous 5 years, HPV vaccination, and chlamydial infection history. Education, occupation, household income, and residence type were entered as codebook-order terms; smoking status and chlamydial infection history were entered using indicator variables. When age at first sexual intercourse was the exposure, model 2 included the remaining prespecified covariates. Model 3 further included current high-risk HPV status. High-risk HPV positivity was evaluated using models 1 and 2. Individual matching and conditional likelihood controlled for age and recruitment time. Gravidity, parity, spontaneous abortions, and induced abortions were modeled separately. Odds ratios (ORs), Wald 95% confidence intervals (CIs), and *P*-values were calculated.

Primary adjusted analyses used complete matched pairs. A pair was excluded when household income was unknown for either member; a sensitivity analysis retained these pairs using the median ordinal income value plus an unknown-income indicator. Age at first pregnancy was analyzed only in pairs in which both members had ever been pregnant. Breastfeeding analyses were restricted to pairs in which both members were parous, with never breastfeeding coded as 0 months among parous women. Nulligravid participants were coded as 0 for gravidity, parity, spontaneous abortions, and induced abortions. Never-users of contraceptive methods were coded as 0 months in cumulative-duration models. User-only duration analyses were restricted to matched pairs in which both members had the relevant exposure; adjusted models included education, smoking status, age at first sexual intercourse, lifetime number of sexual partners, cervical screening frequency, and current hrHPV status.

Model adequacy was summarized using the number of exposure-discordant pairs, the number of estimated parameters, and matched pairs per parameter. Convergence, maximum score gradient, variance inflation factors, scaled condition numbers, and one-step DFBETA diagnostics were examined. Linearity of continuous exposures was assessed with restricted cubic splines using knots at the 5th, 35th, 65th, and 95th percentiles when the number of distinct values was sufficient; flexible and linear specifications were compared using likelihood-ratio tests. Menstrual cycle length and menstrual period duration were additionally examined using prespecified categories. Spearman correlations were calculated among reproductive variables. Multi-parameter exposure, nonlinearity, and interaction terms were evaluated using likelihood-ratio tests.

## 3 Results

### 3.1 Demographic and baseline characteristics

The study population and participant flow are shown in **Figure 1**. Among 681 women assessed for eligibility, 81 were excluded or not enrolled because of pregnancy at enrollment (*n* = 12), severe psychiatric disorders (*n* = 3), incomplete medical records (*n* = 33), missing menstrual or reproductive data (*n* = 15), or refusal to participate (*n* = 18). Finally, 300 women with early-onset cervical cancer and 300 individually matched controls were included in the analysis. Interviews were completed 1–5 days after histologic diagnosis for 33 cases; all 33 were aware of the diagnosis at interview. Demographic, socioeconomic, healthcare utilization, sexual behavior, smoking, infection-related, tumor, and control follow-up characteristics are summarized in **Table 1**. Age and body mass index were similar between groups. Between-group differences were observed for education, screening frequency, HPV vaccination, sexual behavior, high-risk behavior in partners, smoking, passive smoking, chlamydial infection history, current hrHPV status, HPV16 positivity, HPV18 positivity, and persistent hrHPV status. Occupation, household income, residence, and HIV status did not differ between groups. Tumor characteristics among cases and follow-up status among controls are also summarized in **Table 1**. Analytical samples and the handling of structurally inapplicable values are summarized in **Supplementary Table S1**, and exposure definitions and coding are provided in **Supplementary Table S1**. Among 64 current hrHPV-negative cases, 38 had non-squamous histology and six had a positive tissue HPV result. Among 55 current hrHPV-positive/NILM controls, 46 completed final follow-up, with no CIN2+ or cervical cancer identified; nine were lost to follow-up (**Supplementary Table S2**). HPV16 and HPV18 results by histologic type are summarized in **Supplementary Table S2**.

**Table 1:** Demographic, socioeconomic, behavioral, infectious, and clinical characteristics of cases and controls.

Variable	Cases (*n* = 300)	Controls (*n* = 300)	Paired test	Statistic	*P*-value
Demographic, socioeconomic, and healthcare utilization characteristics					
Age at index date, years	30.44 ± 3.08	30.48 ± 3.07	Matching variable	—	—
Body mass index, kg/m²	23.62 ± 3.57	23.75 ± 3.02	Paired *t*-test	*t* = -0.470	0.639
Education level, *n* (%)			Bowker symmetry test	*χ*² = 14.433	0.002
Primary school or less	124 (41.33)	86 (28.67)			
Secondary or vocational school	132 (44.00)	143 (47.67)			
College or above	44 (14.67)	71 (23.67)			
Occupation, *n* (%)			Bowker symmetry test	*χ*² = 7.127	0.068
Professional/managerial (ISCO 1–2)	67 (22.33)	89 (29.67)			
Clerical/service (ISCO 3–5)	98 (32.67)	103 (34.33)			
Manual/physical work (ISCO 6–9)	135 (45.00)	108 (36.00)			
Annual household income, *n* (%)			Bowker symmetry test	*χ*² = 10.834	0.371
<CNY 50,000	88 (29.33)	69 (23.00)			
CNY 50,000–99,999	101 (33.67)	103 (34.33)			
CNY 100,000–199,999	72 (24.00)	80 (26.67)			
≥CNY 200,000	25 (8.33)	39 (13.00)			
Unknown	14 (4.67)	9 (3.00)			
Residence, *n* (%)			Bowker symmetry test	*χ*² = 3.791	0.956
Urban	135 (45.00)	139 (46.33)			
County town	57 (19.00)	49 (16.33)			
Rural	76 (25.33)	86 (28.67)			
Migrant population	20 (6.67)	19 (6.33)			
Unknown	12 (4.00)	7 (2.33)			
Number of screenings in the previous 5 years	1.00 (0.00, 2.00)	2.00 (1.00, 3.00)	Wilcoxon signed-rank test	*W* = 7,945.500	<0.001
HPV vaccination, *n* (%)			McNemar test	*χ*² = 4.024	0.045
No	286 (95.33)	272 (90.67)			
Yes	14 (4.67)	28 (9.33)			
Sexual behavior, smoking, and infection-related characteristics					
Age at first sexual intercourse, years	18.42 ± 2.35	19.51 ± 2.67	Paired t test	*t* = -5.354	<0.001
Lifetime number of sexual partners	3.00 (2.00, 5.00)	2.00 (1.00, 3.00)	Wilcoxon signed-rank test	*W* = 5,581.000	<0.001
New sexual partners in the previous 12 months	0.00 (0.00, 0.00)	0.00 (0.00, 0.00)	Wilcoxon signed-rank test	*W* = 683.000	<0.001
High-risk behavior in partner, *n* (%)			McNemar test	*χ*² = 17.133	<0.001
No	208 (69.33)	253 (84.33)			
Yes	92 (30.67)	47 (15.67)			
Smoking status, *n* (%)			Bowker symmetry test	*χ*² = 13.716	0.003
Never	198 (66.00)	234 (78.00)			
Former	32 (10.67)	28 (9.33)			
Current	70 (23.33)	38 (12.67)			
Passive smoking, *n* (%)			McNemar test	*χ*² = 4.133	0.042
No	198 (66.00)	222 (74.00)			
Yes	102 (34.00)	78 (26.00)			
History of chlamydial infection, *n* (%)			Bowker symmetry test	*χ*² = 8.037	0.045
No	240 (80.00)	227 (75.67)			
Yes	29 (9.67)	21 (7.00)			
Not tested	31 (10.33)	52 (17.33)			
HIV status, *n* (%)			Bowker symmetry test	*χ*² = 3.400	0.183
Negative	292 (97.33)	293 (97.67)			
Positive	3 (1.00)	0 (0.00)			
Not tested	5 (1.67)	7 (2.33)			
High-risk HPV positivity, *n* (%)			McNemar test	*χ*² = 162.814	<0.001
Negative	64 (21.33)	245 (81.67)			
Positive	236 (78.67)	55 (18.33)			
HPV16 positivity, *n* (%)			McNemar test	*χ*² = 123.706	<0.001
Negative	141 (47.00)	284 (94.67)			
Positive	159 (53.00)	16 (5.33)			
HPV18 positivity, *n* (%)			McNemar test	*χ*² = 11.256	<0.001
Negative	266 (88.67)	289 (96.33)			
Positive	34 (11.33)	11 (3.67)			
Persistent high-risk HPV, *n* (%)			Bowker symmetry test	*χ*² = 93.086	<0.001
No	86 (28.67)	129 (43.00)			
Yes	125 (41.67)	18 (6.00)			
Indeterminate	89 (29.67)	153 (51.00)			
Tumor characteristics among cases					
Histologic type, *n* (%)		Not applicable	Case-only description	—	—
Squamous cell carcinoma	214 (71.33)	Not applicable			
Adenocarcinoma	56 (18.67)	Not applicable			
Adenosquamous carcinoma	15 (5.00)	Not applicable			
Neuroendocrine carcinoma	4 (1.33)	Not applicable			
Other rare histologic types	11 (3.67)	Not applicable			
FIGO stage, n (%)		Not applicable	Case-only description	—	—
IA1	44 (14.67)	Not applicable			
IA2	23 (7.67)	Not applicable			
IB1	68 (22.67)	Not applicable			
IB2	49 (16.33)	Not applicable			
IB3	22 (7.33)	Not applicable			
IIA1	24 (8.00)	Not applicable			
IIA2	14 (4.67)	Not applicable			
IIB	39 (13.00)	Not applicable			
IIIC1	17 (5.67)	Not applicable			
Mode of detection, *n* (%)		Not applicable	Case-only description	—	—
Screen-detected	179 (59.67)	Not applicable			
Symptomatic presentation	109 (36.33)	Not applicable			
Incidental detection	12 (4.00)	Not applicable			
Follow-up outcomes among controls					
Final status, *n* (%)	Not applicable		Control-only description	—	—
No lesion	Not applicable	283 (94.33)			
CIN1	Not applicable	6 (2.00)			
Lost to follow-up	Not applicable	11 (3.67)			

Continuous variables are presented as mean ± standard deviation or median (interquartile range) and categorical variables as *n* (%). Descriptive values are shown to two decimal places; test statistics and *P*-values are shown to three decimal places. Between-group inference accounted for 1:1 individual matching. Approximately normally distributed continuous variables were compared using paired *t*-tests, skewed or count variables using Wilcoxon signed-rank tests, binary variables using McNemar tests, and multinomial variables using Bowker symmetry tests. Age at the index date was a matching factor and was not subjected to significance testing. Histologic type, FIGO stage, and mode of detection were described among cases only; control follow-up outcomes were described among controls only. Therefore, no between-group tests were performed for these variables. Genotype-specific results are limited to HPV16 and HPV18.

HPV, human papillomavirus; HIV, human immunodeficiency virus; CIN1, cervical intraepithelial neoplasia grade 1.

### 3.2 Menstrual and reproductive factors

The comparison of menstrual and reproductive characteristics is shown in **Table 2**. Between-group differences were observed for age at menarche, menstrual cycle regularity, pregnancy history, age at first pregnancy, gravidity, parity, spontaneous and induced abortions, breastfeeding history and duration, combined oral contraceptive use and duration, and injectable contraceptive use. Menstrual cycle length, menstrual period duration, menstrual flow category, progestin-only pill use, intrauterine device use, contraceptive implant use, and barrier contraception did not differ between groups.

**Table 2:** Menstrual, reproductive, and contraceptive characteristics of cases and controls.

Variable	Cases (*n* = 300)	Controls (*n* = 300)	Paired test	Statistic	*P*-value
Menstrual characteristics					
Age at menarche, years	12.82 ± 1.66	13.31 ± 1.41	Paired *t*-test	*t* = -3.808	<0.001
Menstrual cycle length, days	28.86 ± 3.74	28.90 ± 3.19	Paired *t*-test	*t* = -0.126	0.900
Menstrual period duration, days	5.27 ± 1.54	5.06 ± 1.26	Paired *t*-test	*t* = 1.919	0.056
Menstrual flow category, *n* (%)			Bowker symmetry test	*χ*² = 2.898	0.408
Light	45 (15.00)	56 (18.67)			
Normal	201 (67.00)	198 (66.00)			
Heavy	54 (18.00)	46 (15.33)			
Menstrual cycle regularity, *n* (%)			McNemar test	*χ*² = 10.537	0.001
Regular (cycle variation <7 days in the previous year)	196 (65.33)	233 (77.67)			
Irregular	104 (34.67)	67 (22.33)			
Pregnancy and reproductive characteristics					
Ever pregnant, *n* (%)			McNemar test	*χ*² = 6.125	0.013
No	33 (11.00)	55 (18.33)			
Yes	267 (89.00)	245 (81.67)			
Age at first pregnancy, years	21.86 ± 2.55	23.18 ± 2.86	Paired *t*-test	*t* = -5.241	<0.001
Gravidity	3.00 (1.00, 3.00)	2.00 (1.00, 2.00)	Wilcoxon signed-rank test	*W* = 6,502.000	<0.001
Parity	2.00 (1.00, 2.00)	1.00 (1.00, 2.00)	Wilcoxon signed-rank test	*W* = 6,227.500	<0.001
Number of spontaneous abortions	0.00 (0.00, 0.00)	0.00 (0.00, 0.00)	Wilcoxon signed-rank test	*W* = 1,702.000	<0.001
Number of medication-induced abortions	0.00 (0.00, 0.00)	0.00 (0.00, 0.00)	Wilcoxon signed-rank test	*W* = 943.500	<0.001
Number of surgical abortions	0.00 (0.00, 0.00)	0.00 (0.00, 0.00)	Wilcoxon signed-rank test	*W* = 1,043.000	<0.001
Total number of induced abortions	0.00 (0.00, 1.00)	0.00 (0.00, 0.00)	Wilcoxon signed-rank test	*W* = 2,443.000	<0.001
Ever breastfed, *n*/*N* (%)			McNemar test	*χ*² = 28.918	<0.001
No	55 (26.57)	12 (5.80)			
Yes	152 (73.43)	195 (94.20)			
Total breastfeeding duration, months	6.00 (4.00, 9.00)	10.00 (7.00, 14.00)	Wilcoxon signed-rank test	*W* = 1,913.000	<0.001
Contraceptive methods					
Ever used combined oral contraceptives, *n* (%)			McNemar test	*χ*² = 16.462	<0.001
No	124 (41.33)	176 (58.67)			
Yes	176 (58.67)	124 (41.33)			
Combined oral contraceptive use status, *n* (%)			Bowker symmetry test	*χ*² = 22.086	0.001
Never used	124 (41.33)	176 (58.67)			
Current use	46 (15.33)	27 (9.00)			
Discontinued ≤12 months ago	43 (14.33)	22 (7.33)			
Discontinued >12 months ago	87 (29.00)	75 (25.00)			
Combined oral contraceptive use duration, months	39.00 (27.00, 63.00)	24.00 (15.00, 39.00)	Wilcoxon signed-rank test	*W* = 611.000	<0.001
Ever used progestin-only pills, *n* (%)			McNemar test	*χ*² = 0.022	0.883
No	275 (91.67)	275 (91.67)			
Yes	25 (8.33)	25 (8.33)			
Progestin-only pill use duration, months	7.00 (5.00, 9.00)	9.50 (8.75, 10.25)	Wilcoxon signed-rank test	*W* = 0.000	1.000
Ever used an intrauterine device, *n* (%)			McNemar test	*χ*² = 3.293	0.070
No	211 (70.33)	188 (62.67)			
Yes	89 (29.67)	112 (37.33)			
Intrauterine device type, *n* (%)			Bowker symmetry test	*χ*² = 8.414	0.135
Never used	211 (70.33)	188 (62.67)			
Copper IUD	81 (27.00)	93 (31.00)			
LNG-IUS	8 (2.67)	15 (5.00)			
Other	0 (0.00)	4 (1.33)			
Intrauterine device use duration, months	30.00 (18.00, 54.00)	42.00 (24.00, 52.50)	Wilcoxon signed-rank test	*W* = 158.500	0.469
Ever used injectable contraceptives, *n* (%)			McNemar test	*χ*² = 6.759	0.009
No	278 (92.67)	293 (97.67)			
Yes	22 (7.33)	7 (2.33)			
Ever used a contraceptive implant, *n* (%)			Exact McNemar test	*b* = 12.000	1.000
No	288 (96.00)	288 (96.00)			
Yes	12 (4.00)	12 (4.00)			
Ever used barrier contraception, *n* (%)			McNemar test	*χ*² = 3.615	0.057
No	233 (77.67)	211 (70.33)			
Yes	67 (22.33)	89 (29.67)			

Continuous variables are presented as mean ± standard deviation or median (interquartile range) and categorical variables as *n* (%). Descriptive values are shown to two decimal places; test statistics and *P*-values are shown to three decimal places. Age at first pregnancy was summarized and tested only in pairs in which both participants had ever been pregnant. Breastfeeding history was summarized among pairs in which both participants had given birth. Breastfeeding duration and contraceptive-use durations in **Table 2** were compared only in matched pairs in which both participants were users. Structurally inapplicable values were not treated as ordinary missing data. Spontaneous and induced abortions are reported separately; induced abortions are further classified as medication-induced or surgical.

IUD, intrauterine device; LNG-IUS, levonorgestrel-releasing intrauterine system.

### 3.3 Multivariable logistic regression analysis

The results of the multivariable logistic regression analysis are presented in **Table 3**. In model 3, the adjusted odds ratios (aORs) were 1.436 per 1-day increase in menstrual period duration (95% CI, 1.044–1.974), 0.717 per 1-year increase in age at first pregnancy (95% CI, 0.561–0.917), 2.959 per pregnancy for gravidity (95% CI, 1.734–5.050), 3.667 per birth for parity (95% CI, 1.886–7.133), 2.672 per induced abortion (95% CI, 1.236–5.773), 0.189 per 12-month increase in total breastfeeding duration (95% CI, 0.064–0.560), and 1.335 per 12-month increase in cumulative combined oral contraceptive use (95% CI, 1.062–1.678). Age at menarche, menstrual cycle length, menstrual flow category, menstrual cycle irregularity, spontaneous abortions, ever breastfeeding, categorical combined oral contraceptive status, progestin-only pill use, intrauterine device use, injectable contraceptive use, contraceptive implant use, and barrier contraception were not statistically significant in model 3. For current hrHPV positivity, the model 2 aOR was 85.176 (95% CI, 21.120–343.517); model 3 was not applicable when current hrHPV positivity was the exposure. The fully adjusted estimates are displayed in **Figure 2**.

**Table 3:** Conditional logistic regression of factors associated with early-onset cervical cancer.

Exposure	Matched pairs M1/M2/M3	Model 1 OR (95% CI)	*P*-value	Model 2 aOR (95% CI)	*P*-value	Model 3 aOR (95% CI)	*P*-value
Age at menarche (per 1-year increase)	300/277/277	0.822 (0.740, 0.913)	<0.001	0.843 (0.723, 0.984)	0.030	0.858 (0.670, 1.098)	0.224
Menstrual cycle length (per 1-day increase)	300/277/277	0.997 (0.953, 1.043)	0.900	1.033 (0.968, 1.102)	0.330	1.003 (0.901, 1.116)	0.958
Menstrual period duration (per 1-day increase)	300/277/277	1.122 (0.996, 1.264)	0.057	1.284 (1.066, 1.547)	0.009	1.436 (1.044, 1.974)	0.026
Light menstrual flow vs. normal	300/277/277	0.778 (0.493, 1.228)	0.281	1.140 (0.544, 2.386)	0.729	0.926 (0.269, 3.191)	0.903
Heavy menstrual flow vs. normal	300/277/277	1.141 (0.742, 1.753)	0.548	1.136 (0.595, 2.167)	0.699	0.940 (0.314, 2.812)	0.912
Irregular menstrual cycles (yes vs. no)	300/277/277	1.860 (1.284, 2.695)	0.001	1.389 (0.804, 2.400)	0.239	1.834 (0.630, 5.333)	0.266
Age at first pregnancy (per 1-year increase)	220/202/202	0.826 (0.763, 0.895)	<0.001	0.792 (0.700, 0.897)	<0.001	0.717 (0.561, 0.917)	0.008
Gravidity (per pregnancy)	300/277/277	1.599 (1.391, 1.839)	<0.001	1.761 (1.430, 2.168)	<0.001	2.959 (1.734, 5.050)	<0.001
Parity (per birth)	300/277/277	1.661 (1.376, 2.006)	<0.001	1.934 (1.452, 2.576)	<0.001	3.667 (1.886, 7.133)	<0.001
Spontaneous abortions (per event)	300/277/277	1.579 (1.059, 2.355)	0.025	1.666 (0.924, 3.005)	0.090	3.319 (0.976, 11.289)	0.055
Induced abortions (per event)	300/277/277	1.792 (1.403, 2.290)	<0.001	1.904 (1.365, 2.657)	<0.001	2.672 (1.236, 5.773)	0.012
Ever breastfed (yes vs. no)	207/189/189	0.173 (0.085, 0.351)	<0.001	0.241 (0.089, 0.654)	0.005	0.298 (0.068, 1.304)	0.108
Total breastfeeding duration (per 12-month increase)	207/189/189	0.190 (0.112, 0.324)	<0.001	0.193 (0.089, 0.420)	<0.001	0.189 (0.064, 0.560)	0.003
Combined oral contraceptives: current use vs. never	300/277/277	2.464 (1.439, 4.220)	0.001	1.987 (0.933, 4.232)	0.075	1.595 (0.408, 6.227)	0.502
Combined oral contraceptives: recent discontinuation vs. never	300/277/277	2.722 (1.554, 4.768)	<0.001	1.901 (0.868, 4.164)	0.108	2.504 (0.723, 8.668)	0.147
Combined oral contraceptives: former use vs. never	300/277/277	1.629 (1.112, 2.386)	0.012	1.367 (0.798, 2.340)	0.255	0.941 (0.338, 2.622)	0.908
Cumulative combined oral contraceptive use (per 12-month increase)	300/277/277	1.322 (1.202, 1.454)	<0.001	1.394 (1.212, 1.602)	<0.001	1.335 (1.062, 1.678)	0.013
Ever used progestin-only pills (yes vs. no)	300/277/277	1.000 (0.561, 1.782)	1.000	1.042 (0.453, 2.399)	0.923	0.456 (0.097, 2.154)	0.322
Cumulative progestin-only pill use (per 12-month increase)	300/277/277	0.839 (0.546, 1.289)	0.422	0.874 (0.450, 1.695)	0.690	0.273 (0.066, 1.137)	0.074
Ever used a copper IUD (yes vs. no)	300/277/277	0.838 (0.598, 1.174)	0.304	0.840 (0.514, 1.371)	0.484	1.459 (0.629, 3.384)	0.379
Ever used an LNG-IUS (yes vs. no)	300/277/277	0.533 (0.226, 1.258)	0.151	0.495 (0.162, 1.505)	0.215	0.402 (0.062, 2.600)	0.338
Cumulative intrauterine device use (per 12-month increase)	300/277/277	0.926 (0.856, 1.001)	0.052	0.890 (0.794, 0.998)	0.046	0.975 (0.817, 1.163)	0.779
Ever used injectable contraceptives (yes vs. no)	300/277/277	3.143 (1.343, 7.357)	0.008	1.260 (0.370, 4.292)	0.712	1.658 (0.248, 11.089)	0.602
Ever used a contraceptive implant (yes vs. no)	300/277/277	1.000 (0.449, 2.226)	1.000	0.723 (0.252, 2.073)	0.546	1.288 (0.185, 8.988)	0.798
Ever used barrier contraception (yes vs. no)	300/277/277	0.694 (0.484, 0.996)	0.048	0.610 (0.364, 1.023)	0.061	0.443 (0.172, 1.137)	0.091
High-risk HPV positivity (yes vs. no)	300/277/—	21.111 (10.818, 41.200)	<0.001	85.176 (21.120, 343.517)	<0.001	—	—

ORs, 95% CIs, and *P*-values are shown to three decimal places. The numbers of informative matched pairs are listed as model 1/model 2/model 3. Model 1: Each exposure was entered separately into a conditional logistic regression stratified by match_set_id. Model 2: The exposure-specific model additionally included prespecified adjustment for education, occupation, household income, residence, smoking, age at first sexual intercourse, lifetime number of sexual partners, screening frequency during the previous 5 years, HPV vaccination, and chlamydial infection history. When age at first sexual intercourse was the exposure, it was not duplicated in the adjustment set. Model 3 further adjusted for high-risk HPV status. Age and recruitment time were controlled through individual matching and the conditional likelihood and were not entered again as conventional covariates. Gravidity, parity, spontaneous abortion, and induced abortion were modeled in separate exposure-specific models. No univariable *P*-value screening or stepwise regression was used. Age at first pregnancy was analyzed only in pairs in which both participants had ever been pregnant; nulligravid participants were coded 0 for gravidity, parity, spontaneous abortion, and induced abortion counts. Breastfeeding-related models were restricted to pairs in which both participants had given birth, with never breastfeeding coded as 0 months. Never-users of contraceptive methods were coded as 0 months in cumulative-duration models. Model 2 and model 3 used complete matched pairs; pairs affected by unknown household income were excluded from the primary adjusted analysis and retained in a sensitivity analysis. When high-risk HPV positivity was the exposure, model 3 was not applicable because it would duplicate the exposure.

OR, odds ratio; aOR, adjusted odds ratio; CI, confidence interval; —, not applicable.

**Figure 2:**
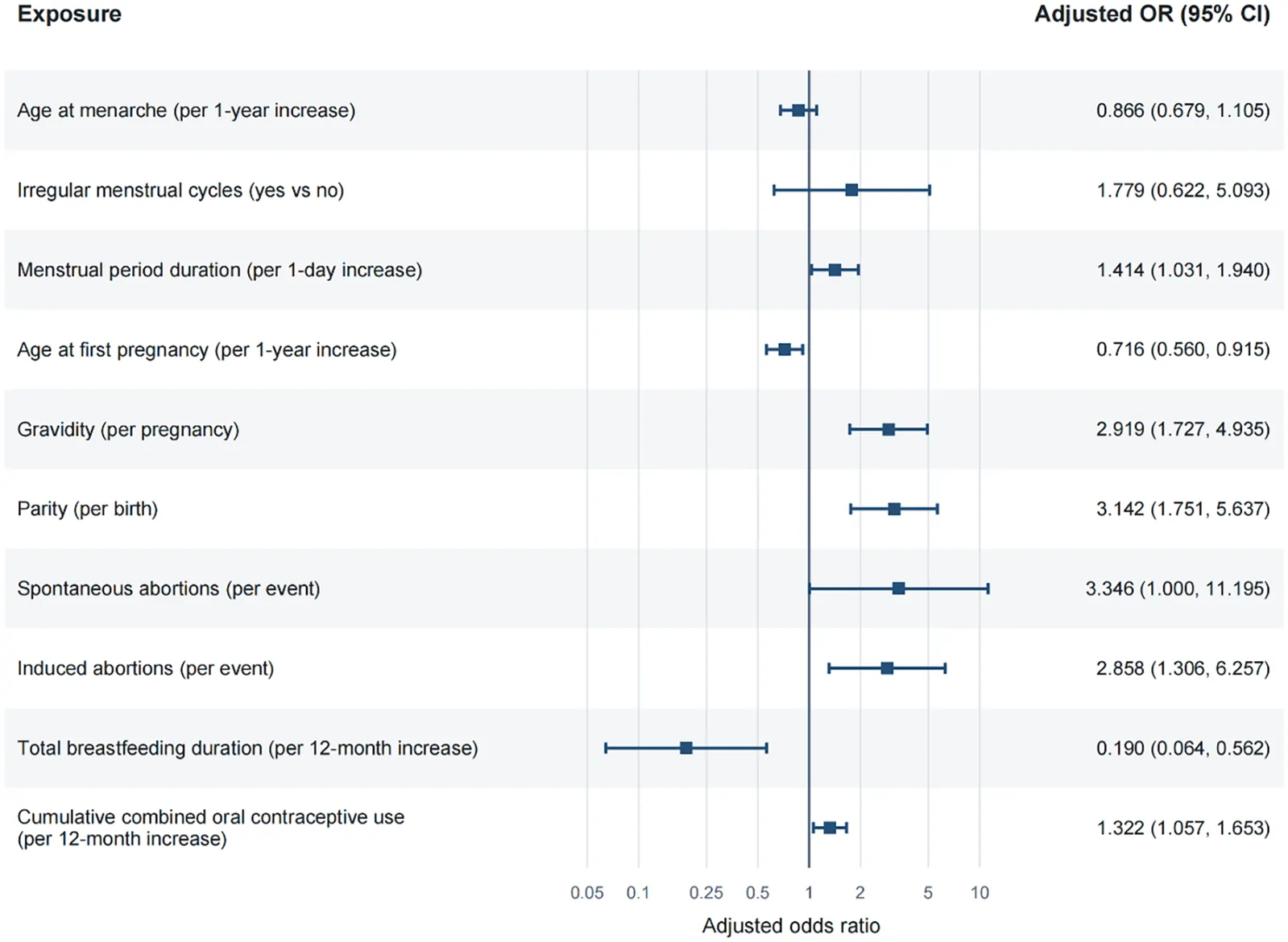
Model 3 estimates for selected menstrual, reproductive, and contraceptive exposures. Squares and horizontal lines denote model 3 adjusted odds ratios and 95% confidence intervals from 1:1 matched conditional logistic regression. The vertical reference line indicates an odds ratio of 1. OR, odds ratio; CI, confidence interval.

Model adequacy, numerical diagnostics, linearity assessments, categorical representations of nonlinear menstrual variables, and key correlations among reproductive variables are reported in **Supplementary Table S3**. Adjusted dose–response associations are shown for age at menarche (**Supplementary Figure S1A**), menstrual cycle length (**Supplementary Figure S1B**), menstrual period duration (**Supplementary Figure S1C**), age at first pregnancy (**Supplementary Figure S1D**), gravidity (**Supplementary Figure S1E**), parity (**Supplementary Figure S1F**), spontaneous abortions (**Supplementary Figure S1G**), total breastfeeding duration (**Supplementary Figure S1H**), and cumulative combined oral contraceptive use (**Supplementary Figure S1I**). The nonlinearity likelihood-ratio *P*-values were 0.013 for menstrual cycle length, <0.001 for menstrual period duration, 0.046 for gravidity, and <0.001 for total breastfeeding duration (**Supplementary Table S3**). Categorical analyses yielded global likelihood-ratio *P*-values of 0.170 for menstrual cycle length and 0.039 for menstrual period duration (**Supplementary Table S3**).

### 3.4 Subgroup and sensitivity analyses

Subgroup estimates and interaction tests are presented in **Table 4**. The 25 interaction tests had unadjusted *P*-values of 0.036–0.911 and false-discovery-rate *q*-values of 0.627–0.911. The sensitivity analyses of the principal associations are presented in **Supplementary Table S4**. Excluding the nine hrHPV-positive/NILM controls lost to follow-up and their matched cases yielded 269 standard-exposure pairs, while excluding the 33 cases interviewed after diagnosis and their matched controls yielded 246 standard-exposure pairs. In these two analyses, respectively, the model 3 *P*-values were 0.026 and 0.003 for menstrual period duration, 0.010 and 0.039 for age at first pregnancy, <0.001 and <0.001 for gravidity, <0.001 and 0.001 for parity, 0.010 and 0.039 for induced abortions, 0.002 and 0.018 for total breastfeeding duration, 0.011 and 0.020 for cumulative combined oral contraceptive use, and 0.137 and 0.056 for spontaneous abortions, respectively. The expanded adjustment model for total breastfeeding duration and the adjusted user-restricted intrauterine-device duration model were not estimable; the progestin-only-pill user-restricted analysis included two matched pairs and was not estimable.

**Table 4:** Exploratory subgroup analyses and formal tests for interaction.

Exposure	Group 1 aOR (95% CI)	*P*-value	Group 2 aOR (95% CI)	*P*-value	*P* for interaction	FDR *q*-value
Education: primary education or below (*n* = 210) versus secondary education or above (*n* = 390)						
Age at menarche (per 1-year increase)	0.926 (0.612, 1.400)	0.716	0.825 (0.587, 1.160)	0.268	0.687	0.881
Irregular menstrual cycles (yes vs. no)	0.999 (0.237, 4.217)	0.999	2.821 (0.742, 10.715)	0.128	0.238	0.766
Spontaneous abortions (per event)	1.571 (0.237, 10.418)	0.640	5.453 (1.070, 27.789)	0.041	0.309	0.766
Induced abortions (per event)	3.625 (1.019, 12.896)	0.047	2.103 (0.816, 5.417)	0.124	0.501	0.881
Cumulative combined oral contraceptive use (per 12-month increase)	1.127 (0.800, 1.587)	0.493	1.473 (1.097, 1.976)	0.010	0.249	0.766
Occupation: manual labour (*n* = 243) versus non-manual labour (*n* = 357)						
Age at menarche (per 1-year increase)	1.025 (0.681, 1.543)	0.906	0.748 (0.512, 1.091)	0.132	0.310	0.766
Irregular menstrual cycles (yes vs. no)	0.984 (0.278, 3.490)	0.981	4.566 (0.967, 21.569)	0.055	0.083	0.694
Spontaneous abortions (per event)	0.641 (0.107, 3.842)	0.627	8.900 (1.668, 47.484)	0.010	0.036	0.627
Induced abortions (per event)	1.616 (0.469, 5.571)	0.447	3.880 (1.485, 10.138)	0.006	0.246	0.766
Cumulative combined oral contraceptive use (per 12-month increase)	1.352 (0.927, 1.972)	0.117	1.440 (1.067, 1.945)	0.017	0.791	0.893
Age at first sexual intercourse: ≤18 years (*n* = 235) versus >18 years (*n* = 365)						
Age at menarche (per 1-year increase)	0.774 (0.509, 1.179)	0.233	0.904 (0.648, 1.260)	0.550	0.593	0.881
Irregular menstrual cycles (yes vs. no)	2.115 (0.470, 9.511)	0.329	1.591 (0.461, 5.489)	0.462	0.740	0.881
Spontaneous abortions (per event)	2.770 (0.387, 19.853)	0.311	4.194 (1.003, 17.542)	0.050	0.719	0.881
Induced abortions (per event)	3.760 (0.786, 17.974)	0.097	2.203 (0.917, 5.292)	0.077	0.551	0.881
Cumulative combined oral contraceptive use (per 12-month increase)	1.376 (0.921, 2.057)	0.119	1.230 (0.963, 1.571)	0.097	0.620	0.881
Smoking status: never smoker (*n* = 432) versus ever smoker (*n* = 168)						
Age at menarche (per 1-year increase)	0.851 (0.645, 1.123)	0.254	0.825 (0.515, 1.322)	0.424	0.911	0.911
Irregular menstrual cycles (yes vs. no)	1.646 (0.544, 4.983)	0.378	4.489 (0.564, 35.748)	0.156	0.362	0.766
Spontaneous abortions (per event)	2.116 (0.565, 7.921)	0.266	7.481 (0.570, 98.212)	0.126	0.354	0.766
Induced abortions (per event)	3.116 (1.327, 7.316)	0.009	1.272 (0.232, 6.985)	0.782	0.368	0.766
Cumulative combined oral contraceptive use (per 12-month increase)	1.360 (1.038, 1.783)	0.026	1.256 (0.904, 1.744)	0.175	0.689	0.881
High-risk HPV status: negative (*n* = 309) versus positive (*n* = 291)						
Age at menarche (per 1-year increase)	0.775 (0.555, 1.080)	0.132	1.007 (0.667, 1.520)	0.973	0.357	0.766
Irregular menstrual cycles (yes vs. no)	1.709 (0.459, 6.355)	0.424	2.055 (0.395, 10.689)	0.392	0.857	0.893
Spontaneous abortions (per event)	4.330 (0.960, 19.523)	0.056	2.216 (0.383, 12.817)	0.374	0.555	0.881
Induced abortions (per event)	2.848 (1.064, 7.626)	0.037	2.429 (0.748, 7.891)	0.140	0.838	0.893
Cumulative combined oral contraceptive use (per 12-month increase)	1.748 (1.179, 2.593)	0.005	1.082 (0.801, 1.461)	0.608	0.050	0.627

Subgroup-specific aORs were obtained as linear combinations from conditional logistic regression models containing the exposure, subgroup indicator, and exposure-by-subgroup interaction. All models were stratified by match_set_id and used the model 3 prespecified adjustment set; the stratifying variable was not duplicated in its original form. *P* for interaction was calculated using a likelihood-ratio test comparing models with and without the interaction term. FDR *q*-values were calculated across the 25 interaction tests using the Benjamini–Hochberg procedure. Differences between subgroups were evaluated using the interaction tests. All 25 FDR *q*-values were ≥0.627. Supplementary statistical methods: All regression analyses used the conditional likelihood for 1:1 individually matched case–control pairs, with match_set_id defining the stratum. Exposure-specific models were prespecified; gravidity, parity, spontaneous abortions, and induced abortions were not entered together. Interaction analyses used product terms and likelihood-ratio tests. Multiple interaction tests were controlled using the Benjamini–Hochberg false-discovery-rate procedure. Continuous exposure assumptions were assessed by comparing the linear specification with a restricted cubic spline or, when values were sparse, a categorical specification. Model diagnostics included convergence, the maximum score gradient, pairs per parameter, maximum variance inflation factor, scaled Hessian condition number, and maximum absolute one-step DFBETA. Sensitivity analyses assessed high-risk HPV adjustment, unknown-income handling, an expanded confounder set, restriction to HPV-associated cancer pairs, restriction to concordant high-risk HPV-positive pairs, common histologic types, exclusion of the most influential matched pair, exclusion of hrHPV-positive/NILM controls lost to follow-up and their matched cases, and exclusion of cases interviewed after diagnosis and their matched controls.

## 4 Discussion

This study provides evidence that menstrual and reproductive factors were associated with the odds of early-onset cervical cancer among women aged ≤35 years. In exposure-specific conditional logistic regression models, menstrual period duration showed a nonlinear association, while younger age at first pregnancy, greater gravidity and parity, more induced abortions, shorter total breastfeeding duration, and longer cumulative combined oral contraceptive use remained associated with early-onset cervical cancer after prespecified adjustment, including adjustment for measured current hrHPV status. These estimates represent associations rather than causal effects, and the category-specific estimates for menstrual period duration were imprecise. The associations identified warrant discussion regarding their biological and epidemiological context, while their clinical implications remain exploratory.

This study demonstrated that cervical cancer cases had an earlier age at menarche and a higher proportion of menstrual irregularity compared to controls. However, neither age at menarche nor menstrual cycle irregularity remained statistically significant in the fully adjusted analysis. Therefore, the observed between-group differences should not be interpreted as independent associations with early-onset cervical cancer. Earlier menarche and menstrual irregularity have nevertheless been associated with cervical cancer or related outcomes in previous studies. A Chinese epidemiological investigation reported that earlier age at menarche is associated with increased cervical cancer risk (25). Li et al. (26) reported that younger age at menarche was associated with increased risk of cervical cancer recurrence. Okyere et al. (27) conducted a study based on Demographic and Health Survey evidence, revealing that earlier menarche was associated with lower cervical cancer screening uptake. Although this finding was not directly related to cervical cancer incidence, it indicates that the relationship between the timing of menarche and cervical-cancer-related outcomes may also involve differences in health behavior and screening participation. Earlier physiological maturation has also been proposed as a potential marker of earlier sexual activity and longer cumulative opportunity for HPV exposure, although these factors were not directly evaluated in the present study. Furthermore, Varghese et al. (13) investigated the US inpatient database and found that women diagnosed with menstrual disorders had significantly higher odds ratios for cervical cancer diagnosis (OR = 1.582). A cross-sectional study conducted among Ethiopian women also revealed that women with a history of irregular menstruation had twice the risk of being diagnosed with cervical precancerous lesions compared to controls (adjusted OR = 2.29) (28). Differences in study populations, menstrual variable definitions, outcomes, and adjustment strategies may contribute to the inconsistency across studies. In the present analysis, menstrual period duration, rather than age at menarche or cycle irregularity, was associated with early-onset cervical cancer. Nevertheless, this relationship was nonlinear, and the individual categorical estimates were imprecise. Menstrual cycle length also showed evidence of nonlinearity without a statistically significant overall association. Thus, constant per-day effects may not adequately characterize the relationships between menstrual characteristics and early-onset cervical cancer.

Regarding reproductive factors, this study demonstrated that the early-onset cervical cancer group exhibited earlier age at first pregnancy and higher numbers of G, P, and A compared to controls. In the adjusted conditional logistic regression analyses, a younger age at first pregnancy and greater gravidity and parity were associated with higher odds of early-onset cervical cancer. These findings are consistent with a previous meta-analysis showing that women with first pregnancy at age 20 or older had a 41% reduced risk of cervical cancer compared to those with first pregnancy before age 20 (29). Another meta-analysis also revealed that gravidity has positive correlation with occurrence of cervical cancer (30). One proposed explanation is that delayed childbearing allows additional time for cervical epithelial maturation, whereas pregnancy-related hormonal and immunological changes may affect cervical susceptibility to persistent HPV infection and neoplastic progression (31, 32). Early pregnancy may reflect early sexual activity, pregnancy-related immunosuppression, or prolonged HPV exposure during vulnerable cervical development (10). Multiple pregnancies and abortions may contribute to cervical trauma, inflammation, and hormonal fluctuations that facilitate malignant transformation (33).

The present analysis further distinguished spontaneous from induced abortions because these events may have different determinants and confounding structures. A higher number of induced abortions remained associated with early-onset cervical cancer, whereas the estimate for spontaneous abortions was borderline and was less stable in sensitivity analyses. These findings should therefore not be interpreted as evidence that abortion itself causes cervical cancer. In addition, because gravidity, parity, abortion history, age at first pregnancy, and breastfeeding are closely related reproductive characteristics, each exposure was evaluated in a separate conditional logistic regression model. The resulting estimates describe exposure-specific associations and should not be interpreted as effects that are mutually independent of all other reproductive variables.

Among women for whom breastfeeding duration was applicable, a longer total breastfeeding duration was associated with lower odds of early-onset cervical cancer, whereas ever having breastfed was not statistically significant. A cohort study conducted among Korean women demonstrated that women with cumulative breastfeeding duration ≥36 months had the lowest cervical cancer risk (16). Compared to the ≥36 months group, women who breastfed for 13–36 months had a 54% increased risk of cervical cancer (OR = 1.54; 95% CI 1.12–2.11), suggesting that prolonged breastfeeding duration may be associated with reduced cervical cancer risk (16). Possible explanations proposed in previous research include lactational amenorrhea, changes in cumulative hormonal exposure, and breastfeeding-related immunological effects. However, another study has suggested that elevated prolactin levels may be a risk factor for cervical cancer (34), indicating that the relationship between prolactin, breastfeeding, and cervical carcinogenesis may be complex and potentially dependent on exposure duration and timing.

The contraceptive analyses showed different patterns according to the specific exposure measure. Categorical combined oral contraceptive use status was not significantly associated with early-onset cervical cancer, whereas longer cumulative combined oral contraceptive use was associated with higher odds of the disease. Currently used oral contraceptives primarily contain estrogen and progestin as active components. The relationship between oral contraceptive use and cervical cancer has been previously established, with relative risks of approximately 1.1 for use less than 5 years, increasing to 1.6 for 5–9 years, and reaching 2.2 for ≥10 years of use (35). More recent epidemiological evidence has also supported the importance of contraceptive formulation and duration when evaluating hormonal contraceptive exposure (36). A potential biological explanation for the association between oral contraceptive use and cervical cancer may involve interactions among estrogen, progestin, and hormone receptors in cervical tissue, thereby affecting the natural history of HPV infection (37). Sex hormones have also been hypothesized to enhance the expression of HPV16 E6 and E7 oncogenes and influence p53-related pathways involved in viral transformation and carcinogenesis (37).

Neither IUD use nor cumulative IUD use duration was significantly associated with early-onset cervical cancer in the adjusted analyses. Previous evidence regarding IUDs and cervical cancer has been inconsistent. A meta-analysis reported that IUD users had an approximately 30% lower risk of cervical cancer (RR = 0.68) and proposed that local immune responses or mechanical effects might contribute to this association (38). However, another study found no reduction in cervical cancer risk and suggested that associations may differ according to the cervical lesion examined and the type of IUD used (39). Differences between copper IUDs and levonorgestrel-releasing intrauterine systems may be relevant because these devices have different hormonal and local biological effects.

Current hrHPV positivity showed the strongest association with early-onset cervical cancer in the multivariable analysis, consistent with multiple previous studies (2, 40). The available high-risk HPV measurement was strongly associated with early-onset cervical cancer in the present analysis. However, HPV status was based on the available clinical measurement rather than repeated longitudinal testing. Consequently, model 3 estimated exposure associations conditional on measured current hrHPV status but did not fully characterize infection persistence, viral load, genotype-specific exposure, or previous clearance; these estimates should not be interpreted as total causal effects. Persistent high-risk HPV infection, rather than a single positive result, is particularly relevant to cervical carcinogenesis (41). Smoking, educational level, occupation, sexual behavior, and healthcare access may also be related to cervical cancer through HPV exposure, screening participation, socioeconomic conditions, or other pathways. Accordingly, menstrual and reproductive characteristics may partly represent broader differences in sexual exposure, healthcare use, or socioeconomic circumstances rather than direct biological effects.

Exploratory subgroup estimates varied across education, occupation, age at first intercourse, smoking, and high-risk HPV strata. However, none of the interaction tests remained statistically significant after false discovery rate correction. Therefore, the analyses provided no statistically robust evidence that the exposure associations differed across the examined subgroups. A statistically significant estimate within one subgroup and a non-significant estimate within another should not itself be interpreted as evidence of effect modification.

The sensitivity analyses, including exclusion of the nine hrHPV-positive/NILM controls lost to follow-up and their matched cases and exclusion of the 33 cases interviewed after diagnosis and their matched controls, were generally consistent with the principal findings for menstrual period duration, age at first pregnancy, gravidity, parity, induced abortions, total breastfeeding duration, and cumulative combined oral contraceptive use. The spontaneous abortion estimate remained imprecise, and the expanded breastfeeding duration model was not estimable. The findings may contribute to etiological research on reproductive and hormonal cofactors in cervical carcinogenesis. HPV vaccination and validated HPV-based screening strategies remain the established approaches to cervical cancer prevention.

Several limitations should be acknowledged. The hospital-based case–control design and retrospective reporting of reproductive events may have introduced recall and selection bias, particularly because controls were recruited from a screened population and 33 cases were interviewed after diagnosis, all of whom were aware of their diagnosis at interview. Excluding these 33 matched pairs yielded generally similar estimates but could not eliminate the possibility of differential recall. The single-center setting may also limit generalizability. HPV status was based on available clinical measurements without comprehensive information on viral load or longitudinal persistence, and genotype-specific analyses were limited to HPV16 and HPV18 because verifiable HPV52/58 source documentation was unavailable. In addition, structurally inapplicable exposures resulted in different numbers of matched pairs across models, and residual confounding from unmeasured sexual, socioeconomic, immunological, and healthcare-related factors cannot be excluded. Therefore, the observed associations should not be interpreted as causal. Future multicenter prospective studies incorporating repeated HPV testing, more detailed reproductive and contraceptive exposure assessment, and histology-specific analyses are needed to confirm these findings. Tissue HPV confirmation was unavailable for 28 current hrHPV-negative cases, and nine current hrHPV-positive/NILM controls were lost to follow-up, leaving some potential for exposure and control status misclassification.

## 5 Conclusions

This individually matched case–control study found that selected menstrual, reproductive, and contraceptive characteristics were associated with the odds of early-onset cervical cancer among women aged ≤35 years after prespecified adjustment. The observed associations were exposure specific, showed different levels of statistical robustness, and did not establish causality or statistically significant effect modification across the examined subgroups. Further prospective studies with detailed longitudinal HPV and reproductive exposure assessment are needed before these characteristics can have any role beyond etiological research.

## Data Availability

The original contributions presented in the study are included in the article/**Supplementary Material**. Further inquiries can be directed to the corresponding author.

## Supplementary material

Supplementary Figure 1 Model 3 adjusted dose-response associations. Panels **(A–I)** correspond to age at menarche, menstrual cycle length, menstrual period duration, age at first pregnancy, gravidity, parity, spontaneous abortions, total breastfeeding duration, and cumulative combined oral contraceptive use, respectively. Solid curves and shaded areas denote Model 3 adjusted odds ratios and 95% confidence intervals; vertical dashed lines denote reference values, horizontal reference lines indicate an odds ratio of 1, and rug marks indicate observed distributions.
